# Association between ketogenic diet and cognitive function in older adults: The mediating role of neutrophil to high-density lipoprotein cholesterol ratio

**DOI:** 10.1097/MD.0000000000047441

**Published:** 2026-01-30

**Authors:** Qiang Shi, Qiu-Yan Hu, Cheng-Zhi Xu, Jun Zhou, Fei Yin

**Affiliations:** a Department of Emergency, Suzhou Ninth People’s Hospital, Suzhou Ninth Hospital Affiliated to Soochow University, Suzhou, China.

**Keywords:** cognitive function, ketogenic diet, neutrophil to high-density lipoprotein cholesterol ratio, NHANES

## Abstract

The ketogenic diet (KD), characterized by high fat and low carbohydrate intake, has shown potential neuroprotective effects, but its association with cognitive function in older adults and the underlying mechanisms remain unclear. This study aimed to investigate the relationship between KD and cognitive function and explore the mediating role of the neutrophil to high-density lipoprotein cholesterol ratio (NHR), a marker of inflammation and lipid metabolism. We conducted a cross-sectional analysis using data from the National Health and Nutrition Examination Survey 2011 to 2014, involving 2166 participants aged ≥ 60 years. Dietary ketogenic ratio (DKR) was calculated to assess KD adherence, and cognitive function was evaluated using the word learning and recall modules of the Consortium to Establish a Registry for Alzheimer Disease, animal fluency test, digital symbol substitution test and summary Z scores. NHR was computed as the ratio of neutrophil count to HDL-C level. Linear regression, restricted cubic splines model, threshold effect analysis, subgroup analysis and mediation analysis were employed to examine associations and mediating effects. Sensitivity analysis was used to check the robustness of the results. Higher DKR was significantly associated with improved cognitive function, Conversely, elevated NHR was negatively correlated with cognitive function, particularly when exceeding a certain threshold. Mediation figure revealed that NHR mediated 4.83% and 4.75% of the association between DKR and digital symbol substitution test scores and Summary Z scores, respectively, and cognitive function. Subgroup analyses indicated that the associations of DKR and summary Z scores were robust across all subgroups. Sensitivity analysis confirmed the robustness of the results. Our findings suggest that KD is significantly associated with cognitive function in older adults, partially through reducing NHR. These results provide insights into the potential mechanisms linking dietary patterns, inflammation, lipid metabolism, and cognitive health. Further prospective and experimental studies are needed to validate these findings.

## 1. Introduction

With the rapid progress of global aging, the incidence rate of cognitive dysfunction and its related diseases is increasing year by year.^[1]^ About 6.9 million Americans aged over 65 years currently have Alzheimer disease (AD), a number that could double to 13.8 million by 2060.^[2]^ A meta-analysis shows that the prevalence of cognitive impairment among elderly people in China is 21%.^[3]^ Cognitive decline not only affects memory and executive function but also exacerbates physical frailty, mobility limitations, and comorbidities.^[4,5]^ Moreover, patients with AD exhibit higher risks of kidney diseases, lung disease, and cardiovascular events, leading to reduced life expectancy.^[6–8]^ Therefore, prevention and early identification of cognitive dysfunction are crucial.

Cognitive impairment is associated with various factors such as inflammation, oxidative stress, dietary patterns, and exposure to pollutants.^[9–11]^ Ketogenic diet (KD) is characterized by high fat and extremely low carbohydrates, and may exert a protective effect by inducing ketosis, stabilizing blood sugar, and optimizing brain energy metabolism.^[12]^ Recent research suggests KD improves cognitive performance in mild cognitive impairment or AD.^[13,14]^ However, there is still a lack of research on the association between KD and cognitive function in the population.

The level of inflammation and blood lipid in the body is another important factor affecting cognitive function. In the body, neutrophils represent the inflammatory state, while high-density lipoprotein cholesterol (HDL-C) reflects the lipid status. In recent years, the neutrophil to high-density lipoprotein cholesterol ratio (NHR) has been proposed as a comprehensive indicator of inflammation and lipid metabolism status. NHR has been proven to effectively predict multiple health outcomes, such as frailty, diabetes, hearing loss and kidney stone.^[15–18]^ However, it is unclear whether NHR plays a mediating role in the association between KD and cognitive function.

To address this gap, we conducted a large-scale cross-sectional study using the National Health and Nutrition Examination Survey (NHANES) data to investigate the association between KD and cognitive function and the mediating role of NHR in American adults. We hope to reveal the association between KD and cognitive function and its potential mechanisms, thereby providing clues from a dietary perspective for the prevention and treatment of cognitive impairment.

## 2. Methods

### 2.1. The study design and population

The NHANES, administered by the U.S. Centers for Disease Control and Prevention (CDC), represents a comprehensive nationwide study designed to assess population health status and related influencing factors. This CDC-led program has received formal approval from the National Center for Health Statistics Institutional Review Board, ensuring adherence to ethical research standards.

**Figure 1.**
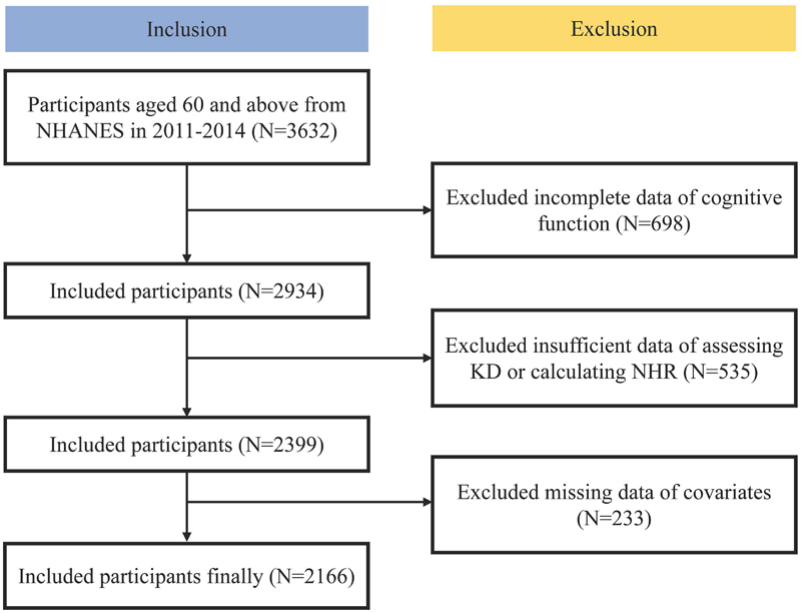
Screening flow of participants.

This study analyzed data from the NHANES 2011 to 2014, initially including 3632 participants aged ≥60 years. The exclusion criteria were applied sequentially: 698 participants with incomplete cognitive function assessments were excluded, retaining 2934 individuals; 535 participants lacking sufficient data on KD adherence or NHR calculations were further excluded, resulting in 2166 eligible participants; and finally, 233 participants with missing covariate data were removed. After these exclusions, the final analytical sample comprised 2166 older adults (Fig. 1).

### 2.2. Assessment of KD

Dietary intake data were collected using 2 separate 24-hour dietary recall assessments. The initial recall involved in-person interviews conducted by trained research staff, followed by a subsequent telephone-administered recall conducted 3 to 10 days later. The dietary intake in this study was the average of the intake from 2 surveys.^[19]^

The dietary ketogenic ratio (DKR) was developed to characterize nutritional patterns capable of inducing ketosis. This metric was formulated through systematic evaluation of the ketogenic and antiketogenic characteristics inherent in macronutrient profiles. The DKR for macronutrients was calculated using the following formula: (0.9 × fat grams + 0.46 × protein grams)/ (0.1 × fat grams + 0.58 × protein grams + net carbohydrate grams). The larger the DKR, the greater the ability of dietary patterns to promote nutritional ketosis.^[20]^

### 2.3. Assessment of cognitive function

The word learning and recall modules of the Consortium to Establish a Registry for AD (CERAD), animal fluency test (AFT), and digital symbol replacement test (DSST) were used to measure cognitive function in this study. CERAD Word Learning subtest can evaluate both immediate and delayed learning abilities. AFT) asks participants to name as many animals as possible within 60 seconds; the semantic fluency score reflects language and executive functions. DSST is a timed paper-and-pencil task primarily measures processing speed, sustained attention and executive function. In addition, we calculated the summary Z scores by normalizing the 3 test scores separately and adding them.^[21]^ The higher the 4 scores, the better the cognitive ability.

### 2.4. Calculation of NHR

NHR was calculated as the ratio of the neutrophil count (in units of 10^3^ cells/μL) to the HDL-C level (in mmol/L). Beckman Coulter DxH 800 analyzer calculates whole blood count, Roche Modular P and Cobas 6000 systems accurately measure HDL-C levels using enzymatic assay.^[22]^

### 2.5. Covariates

Covariates were based on prior knowledge and literature reports, including variables that are known or suspected to be associated with KD compliance and cognitive function. They encompassed demographic, behavioral, and disease-related factors (see Table S1, Supplemental Digital Content, https://links.lww.com/MD/R268 for complete categorization).

### 2.6. Statistical analysis

Statistical analyses were conducted using R software (version 4.5.0), with a 2-tailed alpha level of 0.05 established as the threshold for significance. Examination weights from the mobile examination center were applied in our analyses. Since we include 2 NHANES cycles, the survey weight is the mean of mobile examination center weight. Our analysis began by dividing the research subjects into 4 groups based on the quartiles of DKR and comparing characteristics between the 4 groups, presenting continuous variables as means ± standard deviations (analyzed by t-test) and categorical variables as numbers and proportions (assessed using chi-square tests).^[23]^ We used natural logarithm (Ln) transformation to NHR to normalize it distribution.^[24]^ Using linear regression model, we evaluated associations between DKR, NHR (analyzed continuously and categorically) and cognitive function and calculated regression coefficient β with 95% confidence intervals (CI) after adjusting for all covariates.^[25,26]^ To assess possible nonlinear dose-response relationships, we employed restricted cubic splines regression model.^[27]^ If there is a significant nonlinear correlation, 2-piecewise linear regression model based on threshold analysis was employed.^[28]^ Subgroup analysis assessed interaction effects of DKR and covariates by using likelihood ratio tests.^[29]^ Mediation analysis was applied to evaluate the mediation role of NHR in association between DKR and cognitive function, calculating total effect, direct effect (DE), and indirect effect (i.e.) through 500 bootstrap iterations.^[30]^ Finally, we used depression as an additional covariate and established a sensitivity analysis to test the robustness of the results. Depression was diagnosed based on the Patient Health Questionnaire (PHQ-9) consisting of 9 items. PHQ-9 score ≥ 10 is defined as depressive symptoms.

## 3. Results

### 3.1. Characteristics of study participants

Table 1 displayed the characteristics of study participants. This study included 2166 elderly individuals with an average age of 69.382 years, of whom 51.8% were female. Participants in the higher DKR group tended to be younger, female, Non-Hispanic White, with higher education levels, with higher BMI, nonsmokers, nondrinkers, with higher prevalence of diabetes and with lower energy intake. Importantly, the higher DKR group had significantly higher CERAD scores, AFT scores, DSST scores and summary Z scores.

**Table 1:** Characteristics of participants grouped by quartiles of DKR in the NHANES 2011 to 2014.

Variables	Total	DKR				*P*
		Q1 (≤0.31)	Q2 (0.31–0.37)	Q3 (0.37–0.45)	Q4 (>0.45)	–
n	2166	542	541	541	542	–
Age, mean (SD)	69.382 (6.741)	69.738 (6.666)	70.224 (7.115)	68.974 (6.738)	68.594 (6.321)	<.001
Gender, n (%)						
Male	1044 (48.2)	230 (42.4)	255 (47.1)	280 (51.8)	279 (51.5)	.006
Female	1122 (51.8)	312 (57.6)	286 (52.9)	261 (48.2)	263 (48.5)	
Race, n (%)						
Non-Hispanic White	1131 (52.2)	224 (41.3)	289 (53.4)	303 (56.0)	315 (58.1)	<.001
Non-Hispanic Black	481 (22.2)	136 (25.1)	117 (21.6)	107 (19.8)	121 (22.3)	
Other Race - Including Multi-Racial	554 (25.6)	182 (33.6)	135 (25.0)	131 (24.2)	106 (19.6)	
Education, n (%)						
Less than high school	485 (22.4)	169 (31.2)	118 (21.8)	98 (18.1)	100 (18.5)	<.001
High school grad/GED or equivalent	519 (24.0)	130 (24.0)	135 (25.0)	128 (23.7)	126 (23.2)	
Higher than high school	1162 (53.6)	243 (44.8)	288 (53.2)	315 (58.2)	316 (58.3)	
PIR, n (%)						
≤1.3	596 (27.5)	191 (35.2)	162 (29.9)	111 (20.5)	132 (24.4)	<.001
1.3–3.5	855 (39.5)	202 (37.3)	211 (39.0)	229 (42.3)	213 (39.3)	
>3.5	715 (33.0)	149 (27.5)	168 (31.1)	201 (37.2)	197 (36.3)	
Marital status, n (%)						
Married/living with partner	1282 (59.2)	295 (54.4)	315 (58.2)	336 (62.1)	336 (62.0)	.043
Widowed/divorced/separated	765 (35.3)	212 (39.1)	204 (37.7)	176 (32.5)	173 (31.9)	
Never married	119 (5.5)	35 (6.5)	22 (4.1)	29 (5.4)	33 (6.1)	
BMI, n (%)						
<25 kg/m^2^	564 (26.0)	171 (31.5)	142 (26.2)	137 (25.3)	114 (21.0)	.008
25–30 kg/m^2^	757 (34.9)	181 (33.4)	192 (35.5)	193 (35.7)	191 (35.2)	
≥30 kg/m^2^	845 (39.0)	190 (35.1)	207 (38.3)	211 (39.0)	237 (43.7)	
Smoking status, n (%)						
Nonsmokers	1102 (50.9)	238 (43.9)	264 (48.8)	277 (51.2)	323 (59.6)	<.001
Smokers	1064 (49.1)	304 (56.1)	277 (51.2)	264 (48.8)	219 (40.4)	
Drinking status, n (%)						
Non drinkers	1508 (69.6)	319 (58.9)	367 (67.8)	404 (74.7)	418 (77.1)	<.001
Drinkers	658 (30.4)	223 (41.1)	174 (32.2)	137 (25.3)	124 (22.9)	
Diabetes, n (%)						
Yes	504 (23.3)	110 (20.3)	117 (21.6)	128 (23.7)	149 (27.5)	.03
No	1662 (76.7)	432 (79.7)	424 (78.4)	413 (76.3)	393 (72.5)	
Hypertension, n (%)						
Yes	1356 (62.6)	342 (63.1)	329 (60.8)	341 (63.0)	344 (63.5)	.798
No	810 (37.4)	200 (36.9)	212 (39.2)	200 (37.0)	198 (36.5)	
Activity, n (%)						
Yes	1061 (49.0)	248 (45.8)	272 (50.3)	280 (51.8)	261 (48.2)	.218
No	1105 (51.0)	294 (54.2)	269 (49.7)	261 (48.2)	281 (51.8)	
Energy intake (kal, mean [SD])	1832.363 (675.122)	1658.757 (630.616)	1830.238 (660.409)	1958.713 (675.030)	1881.973 (698.241)	<.001
CERAD scores, mean (SD)	25.286 (6.385)	24.443 (6.402)	24.614 (6.485)	25.810 (6.335)	26.279 (6.142)	<.001
AFT Z scores, mean (SD)	17.000 (5.445)	16.129 (5.349)	16.726 (5.483)	17.270 (5.302)	17.873 (5.505)	<.001
DSST scores, mean (SD)	47.456 (16.704)	43.450 (16.301)	46.675 (17.026)	48.872 (15.819)	50.827 (16.783)	<.001
Summary Z scores, mean (SD)	0.110 (0.964)	-0.120 (0.926)	0.041 (0.983)	0.199 (0.923)	0.321 (0.967)	<.001
NHR, mean (SD)	3.254 (1.742)	3.239 (1.795)	3.291 (1.745)	3.234 (1.774)	3.253 (1.657)	.950

AFT = animal fluency test, BMI = body mass index, CERAD = Consortium to Establish a Registry for Alzheimer Disease, DKR = dietary ketogenic ratio, DSST = digital symbol substitution test, NHANES = National Health and Nutrition Examination Survey, NHR = neutrophil to high-density lipoprotein cholesterol ratio, PIR = poverty income ratio, SD = standard deviation.

### 3.2. The associations of DKR and NHR with cognitive function

Table 2 showed the association of DKR and NHR with cognitive function in linear regression models considering all covariates. For DKR, per unit in DKR increase was associated with 3.15, 8.39 and 0.52 ‌increases in CERAD scores, DSST scores and Summary Z scores (β: 3.15, 95% CI: 0.86–5.44, *P* = .007; β: 8.39, 95% CI: 3.46–13.31, *P* = .001; β: 0.52, 95% CI: 0.24–0.80, *P* <.001). Analysis by group of DKR showed that the CERAD scores, DSST scores and Summary Z scores significantly increased with higher DKR (All *P* for trend <.05). Compared with the lowest DKR group, the Q4 group had higher CERAD scores, DSST scores and Summary Z scores (β: 0.96, 95% CI: 0.25–1.66, *P* = .008; β: 2.88, 95% CI: 1.36–4.40, *P* < .001; β: 0.17, 95% CI: 0.09–0.26, *P* <.001). The above results show that higher DKR is significantly correlated with better cognitive function.

**Table 2:** The associations of DKR, NHR and cognitive function in linear regression models.

	CERAD scores	*P*	AFT scores	*P*	DSST scores	*P*	Summary Z scores	*P*
	β (95% CI)		β (95% CI)		β (95% CI)		β (95% CI)	
DKR								
Continuous	3.15 (0.86–5.44)	.007	1.34 (−0.59 to 3.27)	.173	8.39 (3.46–13.31)	.001	0.52 (0.24–0.80)	<.001
Q1	Reference		Reference		Reference		Reference	
Q2	−0.13 (−0.82 to 0.56)	.706	−0.06 (−0.64 to 0.52)	.83	1.02 (−0.46 to 2.50)	.177	0.03 (−0.05 to 0.12)	.442
Q3	0.54 (−0.16 to 1.24)	.131	−0.12 (−0.71 to 0.47)	.697	0.84 (−0.67 to 2.35)	.273	0.05 (−0.04 to 0.14)	.246
Q4	0.96 (0.25–1.66)	.008	0.48 (−0.12 to 1.07)	.114	2.88 (1.36–4.40)	<.001	0.17 (0.09–0.26)	<.001
* P* for trend	0.002		0.104		< 0.001		< 0.001	
Ln-NHR								
Continuous	−0.46 (−0.98 to 0.06)	.08	−0.38 (−0.82 to 0.05)	.084	−1.65 (−2.76 to −0.53)	.004	−0.10 (−0.16 to −0.04)	.002
Q1	Reference		Reference		Reference		Reference	
Q2	−0.69 (−1.39 to 0.01)	.054	0.11 (−0.48 to 0.69)	.725	0.1 (−1.41 to 1.60)	.901	−0.02 (−0.11 to 0.07)	.655
Q3	−0.62 (−1.34 to 0.10)	.093	−0.09 (−0.70 to 0.52)	.772	−0.83 (−2.38 to 0.73)	.296	−0.06 (−0.15 to 0.03)	.172
Q4	−0.83 (−1.59 to −0.07)	.033	−0.72 (−1.36 to −0.08)	.027	−2.74 (−4.38 to −1.11)	.001	−0.17 (−0.27 to −0.08)	<.001
* P* for trend	0.044		0.025		< 0.001		< 0.001	

Models were adjusted for age, gender, race, education, PIR, marital status, BMI, smoking status, drinking status, diabetes, hypertension, activity and energy intake.

BMI = body mass index, CERAD = Consortium to Establish a Registry for Alzheimer Disease, DKR = dietary ketogenic ratio, NHR = neutrophil to high-density lipoprotein cholesterol ratio, PIR = poverty income ratio.

For NHR, per unit in Ln-NHR increase was associated with 1.65 and 0.1 reductions in DSST scores and Summary Z scores (β: −1.65, 95% CI: −2.76 to −0.53, *P* = .004; β: −0.10, 95% CI: −0.16 to −0.04, *P* = .002). Analysis by group of Ln-NHR showed that the 4 types of scores significantly decreased with higher Ln-NHR (All *P* for trend <.05). Compared with the lowest NHR group, the Q4 group all had lower scores (β: −0.83, 95% CI: −1.59 to −0.07, *P* = .033; β: −0.72, 95% CI: −1.36 to −0.08, *P* = .027; β: −2.74, 95% CI: −4.38 to −1.11, *P* = .001; β: −0.17, 95% CI: −0.27 to −0.08, *P* < .001). The above results show that higher NHR is significantly correlated with worse cognitive function.

**Figure 2.**
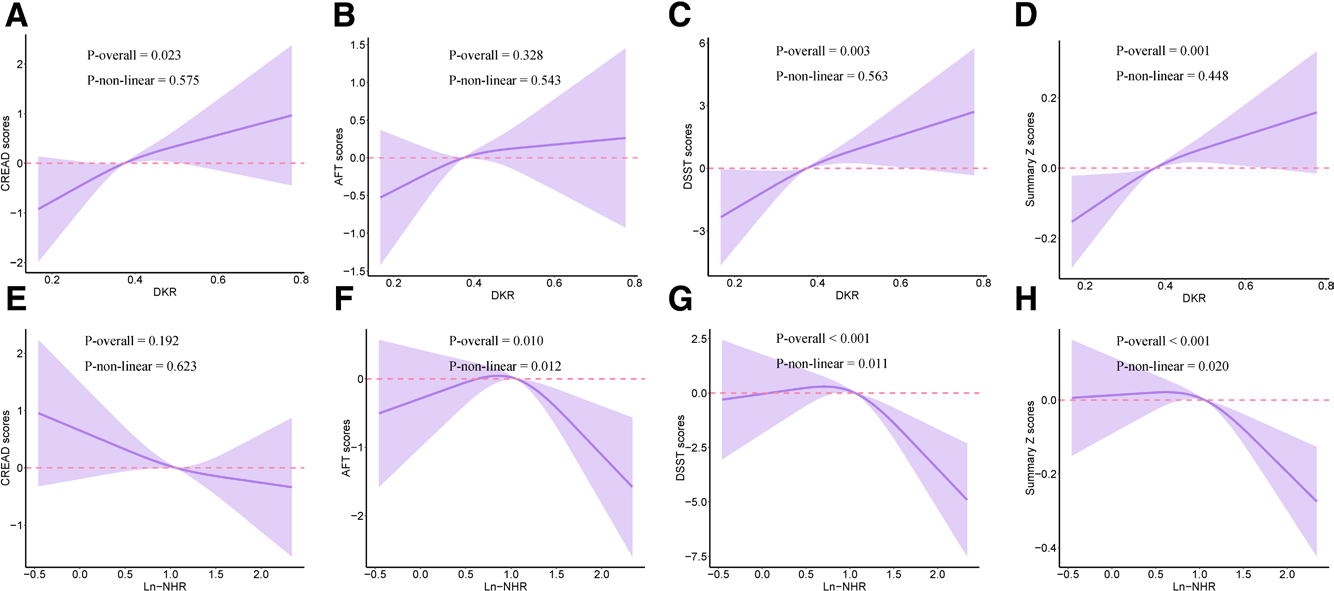
The associations of DKR, NHR and cognitive function in RCS regression models. Models were adjusted for age, gender, race, education, PIR, marital status, BMI, smoking status, drinking status, diabetes, hypertension, activity and energy intake. (A) DKR and CERAD scores; (B) DKR and AFT scores; (C) DKR and DSST scores; (D) DKR and Summary Z scores; (E) NHR and CERAD scores; (F) NHR and AFT scores; (G) NHR and DSST scores; (H) NHR and Summary Z scores. AFT = animal fluency test, BMI = body mass index, CERAD = Consortium to Establish a Registry for Alzheimer Disease, DKR = dietary ketogenic ratio, DSST = digital symbol substitution test, NHR = neutrophil to high-density lipoprotein cholesterol ratio, PIR = poverty income ratio, RCS = restricted cubic splines.

Subsequently, we established restricted cubic splines regression models to investigate the nonlinear relationship between DKR, NHR and cognitive function (Fig. 2). For DKR, the models demonstrated linear positive correlations between DKR, CERAD scores, DSST scores and summary Z scores (All *P* for overall < 0.05 and All *P* for nonlinear >.05). For NHR, the models demonstrated reverse L-shape correlations between Ln-NHR, AFT scores, DSST scores and Summary Z scores after considering all covariates (All *P* for overall <.05 and All *P* for nonlinear <.05). The results show that DKR is significantly positively correlated with CERAD, DSST and summary Z scores while NHR has a significant positive correlation with DSST and summary Z scores, and may have a nonlinear correlation with AFT scores.

After that, we established 2-piecewise linear regression models of NHR and cognitive function based on threshold analysis (Table 2). For AFT scores and Ln-NHR, significant positive correlation was observed when Ln-NHR ≤ 0.168 (β = 2.481, 95% CI: 0.438–4.524, *P* <.001) and significant negative correlation was observed when Ln-NHR > 0.168 (β = −0.693, 95% CI: −1.179 to −0.207, *P* = .005). For DSST scores and Ln-NHR, significant negative correlation was observed when Ln-NHR >0.998 (β = −3.969, 95% CI: −5.908 to −2.029, *P* <.001) and no significant correlation was observed when Ln-NHR ≤0.998. For Summary Z scores and Ln-NHR, significant negative correlation was observed when Ln-NHR > 0.899 (β = −0.206, 95% CI: −0.308 to −0.104, *P* <.001) and no significant correlation was observed when Ln-NHR ≤0.899. The log likelihood ratio test of each threshold effect analyses indicated that the 2-piecewise regression models were superior to the 1-piecewise regression models (All *P* for Log likelihood ratio test <.05) (Table 3).

**Table 3:** Two-piecewise linear regression models of NHR and cognitive function.

Ln-NHR	β (95% CI)	*P*
AFT scores		
≤0.168	2.481 (0.438–4.524)	.017
>0.168	−0.693 (−1.179 to −0.207)	.005
Log likelihood ratio test	–	.005
DSST scores		
≤0.998	0.656 (−1.274 to 2.586)	.505
>0.998	−3.969 (-5.908 to −2.029)	<.001
Log likelihood ratio test	–	.004
Summary Z scores		
≤0.899	0.037 (−0.085 to 0.159)	.550
>0.899	−0.206 (−0.308 to −0.104)	<.001
Log likelihood ratio test	–	.009

Models were adjusted for age, gender, race, education, PIR, marital status, BMI, smoking status, drinking status, diabetes, hypertension, activity and energy intake.

AFT = animal fluency test, BMI = body mass index, CI = confidence interval, DSST = digital symbol substitution test, NHR = neutrophil to high-density lipoprotein cholesterol ratio, PIR = poverty income ratio.

### 3.3. Subgroup analysis of DKR and cognitive function

**Figure 3.**
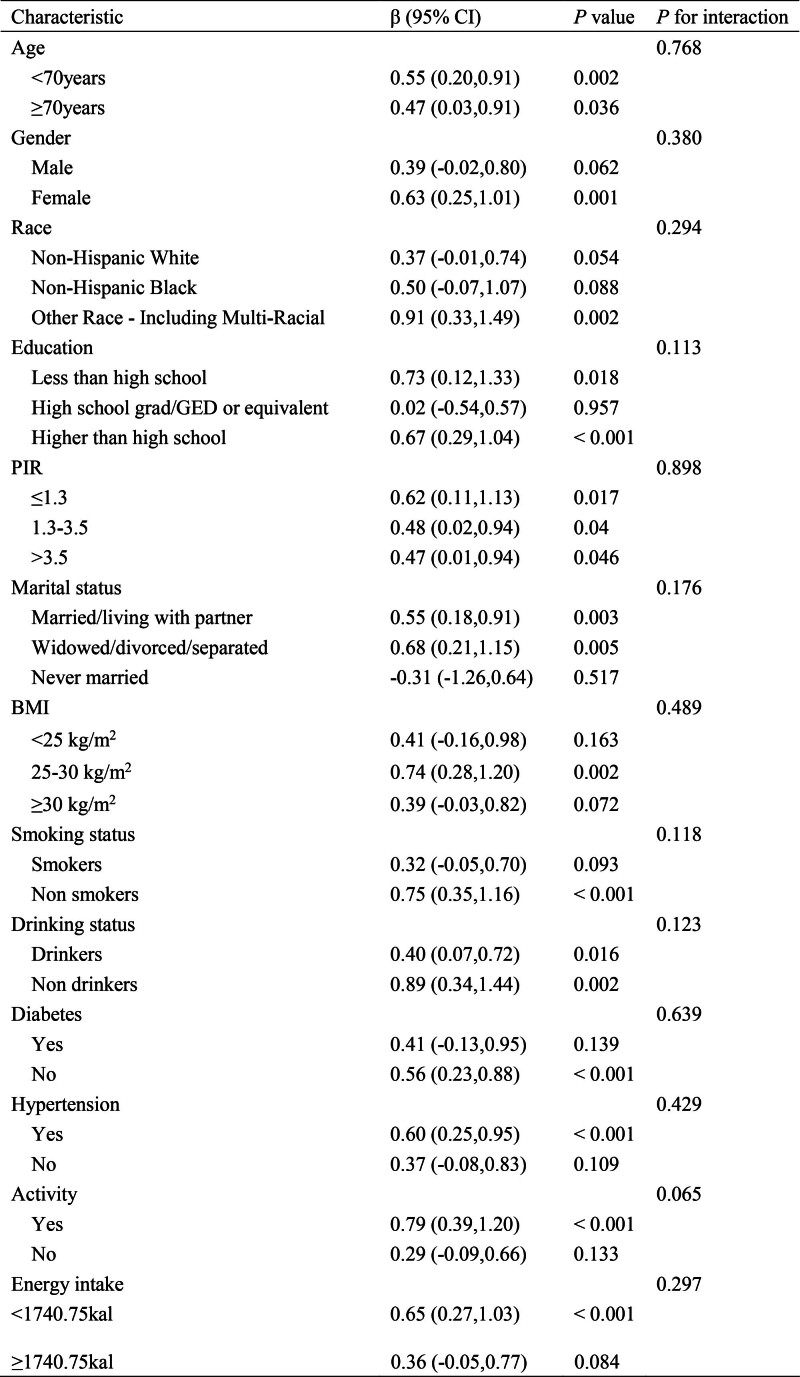
Subgroup analysis of associations of KD and cognitive function. Models were adjusted for age, gender, race, education, PIR, marital status, BMI, smoking status, drinking status, diabetes, hypertension, activity and energy intake. BMI = body mass index, KD = ketogenic diet, PIR = poverty income ratio.

We further evaluated the potential interaction effect of DKR and subgroup variables on Summary Z scores in subgroup analysis (Fig. 3). Age was divided into 2 groups: those under 70 years old and those over 70 years old, and energy intake was divided into 2 groups based on the median. The positive associations between DKR and summary Z scores was consistently observed across nearly all subgroups, corroborating our initial analytical findings. Notably, no significant moderating effects were detected among the examined covariates, demonstrating the robustness of this relationship across diverse population strata (All *P* for interaction >.05).

### 3.4. The mediating role of NHR

**Figure 4.**
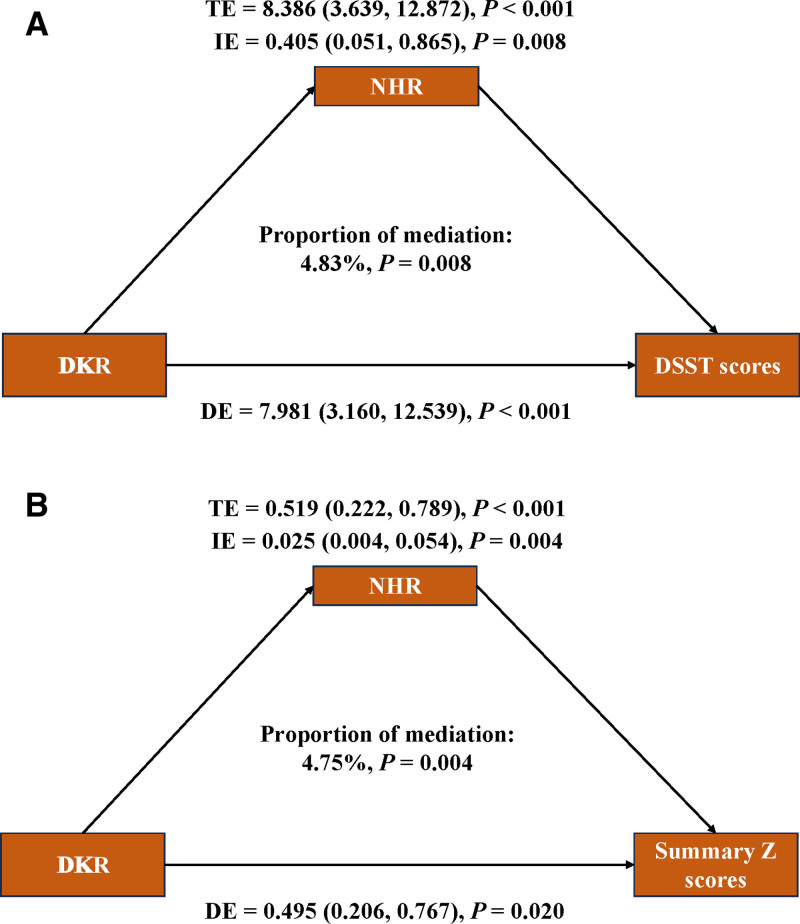
Mediation analysis of exploring the mediating effect of NHR. Models were adjusted for age, gender, race, education, PIR, marital status, BMI, smoking status, drinking status, diabetes, hypertension, activity and energy intake. (A) Mediating effect of NHR in the association of DKR and DSST scores; (B) mediating effect of NHR in the association of DKR and summary Z scores. BMI = body mass index, DKR = dietary ketogenic ratio, DSST = digital symbol substitution test, NHR = neutrophil to high-density lipoprotein cholesterol ratio, PIR = poverty income ratio.

Furthermore, mediation analysis was employed to explore the mediating effect of NHR (Fig. 4). In 2 mediation models, total effect, i.e. and DE were all obvious (*P* <.001). Specifically, Ln-NHR mediated the associations between DKR and DSST scores and DKR and summary Z scores, explaining 4.83% and 4.75% of the corresponding association (Both *P* <.05). These findings suggested that DKR improved cognitive function by lowering NHR.

### 3.5. Sensitivity analysis

In sensitivity analysis, we additionally adjusted for depression. The results showed that the positive correlation between DKR and cognitive function was robust (see Table S2, Supplemental Digital Content, https://links.lww.com/MD/R268).

## 4. Discussion

In summary, this study investigated the association between KD and cognitive function using nationally representative data from the NHANES 2011 to 2014, while also examining the mediating role of NHR in this association. Our results demonstrated DKR was significantly positively with cognitive function, whereas NHR above a certain threshold showed a negative relationship with cognitive function. Finally, mediation analysis confirmed that NHR reduction partially explained the positive association of DKR and cognitive function. The study provides compelling evidence supporting a association between KD and better cognitive function in older U.S. adults and potential mechanisms, which provides clues for improving cognitive function in the elderly population from a dietary perspective and has certain public health significance.

The essence of KD is extremely low carbohydrate and high fat intake and forcing the body to use more fat for energy instead of carbohydrates, aimed at inducing the production of ketone bodies (KET).^[31]^ The positive association of this dietary pattern and the nervous system is receiving increasing attention. Previous studies have confirmed our conclusion that KD can improve cognitive dysfunction. A clinical trial involving 96 obese participants showed that cognitive function improvement was most significant in the study subjects who underwent KD intervention.^[32]^ Another randomized crossover trial showed that KD improved cerebral blood flow and brain-derived neurotrophic factor in healthy individuals.^[33]^ Another study revealed that after participants adopted a modified Mediterranean KD, their cerebrospinal fluid amyloid β-protein (Aβ) increased and tau protein decreased.^[34]^ Several experimental studies still confirm our viewpoint. Mice fed a KD diet showed significant improvements in learning and memory tests, as well as reduced Aβ deposition in the hippocampus and amygdala. KD diet can also reduce depressive or anxious behavior in mice.^[35]^ However, there is currently a lack of research exploring the association between KD and neurological outcomes in large-scale populations. In a word, we conducted a large-scale cross-sectional study and further confirmed the positive association of KD and cognition function.

The mechanism by which KD cognitive function improves has not been fully revealed. This may involve multiple aspects, such as anti-inflammatory, antioxidant, energy utilization, and improving gut microbiota. KD inhibits the expression of inflammatory factors Interleukin-1β (IL-1β), tumor necrosis factor-α (TNF-α), and cyclooxygenase-2 (COX-2) in mice, and promote hippocampal neurogenesis.^[36]^ In addition, KET stimulate mitochondrial biosynthesis, improve mitochondrial function, alleviate oxidative stress, and thereby improve the nervous system.^[37]^ As is known to all, aging brain often accompanies glucose metabolism disorders. KET can bypass insulin resistance and provide efficient energy to neurons, ensuring the normal functioning of the nervous system.^[38]^ Jiang et al proved that KD alters gut microbiota composition, increases beneficial bacteria producing short chain fatty acids, and indirectly improves cognitive function through the gut brain axis.^[39]^ In this study, we found that NHR mediated the improvement effect of KD on cognitive function and showed detrimental effects emerging only when NHR exceeded a critical threshold. This suggests that NHR-associated cognitive impairment may occur only under conditions of severe inflammatory-lipid dysregulation. The increase in neutrophil count is associated with systemic inflammation, while HDL-C has anti-inflammatory properties.^[40]^ Beyond the threshold, excessive neutrophil activation may overwhelm HDL-C’s anti-inflammatory and antioxidant capacity, leading to neurotoxicity.^[41]^ Our study was a large-scale epidemiological study. Therefore, its biological implications are limited. Although the mediating effect of NHR was relatively small, it reached statistical significance. Thus, NHR can be considered a statistically significant mediator between KD and cognitive function. More research is needed in the future to validate our conclusions.

This study has several notable strengths. First, our study is the first to explore the relation between KD and cognitive function in American older people identify the NHR as both a mediator and threshold-dependent risk factor for cognitive decline, offering new insights into diet factor and inflammation-lipid interactions in brain health. Second, the use of nationally representative data from the NHANES enhances the generalizability of our findings. Third, we used multiple statistical methods to comprehensively explore the relationships between variables and increase the reliability of our conclusions.

Despite these advantages, some limitations are inevitable. First, this study is a cross-sectional study, and the determination of causal relationships is limited. Second, KD can be evaluated through 24-hour recall, which may be affected by measurement bias and recall bias, although the average of 2 recalls can improve accuracy. The effectiveness of DKR as a true indicator of ketogenic compliance is uncertain, and future studies should be combined with blood testing to verify it. Third, despite meticulous adjustment, residual confounding cannot be ruled out due to unmeasured factors like genetic predisposition. Finally, the population of this study is the American population, so it is necessary to be cautious when extrapolating the research conclusions to other regions.

## 5. Conclusion

Our study provides convincing evidence for the significant correlation KD and better cognitive function, partially mediated by NHR. Future research should validate these findings in prospective cohorts and experimental studies.

## Acknowledgments

We are grateful to the National Health and Nutrition Examination Survey (NHANES) staff and participants for their valuable contributions, particularly for providing data licenses.

## Author contributions

**Conceptualization:** Qiang Shi.

**Data curation:** Qiang Shi, Jun Zhou.

**Formal analysis:** Qiang Shi, Jun Zhou, Fei Yin.

**Investigation:** Qiu-Yan Hu, Jun Zhou, Fei Yin.

**Methodology:** Qiu-Yan Hu.

**Project administration:** Qiu-Yan Hu, Fei Yin.

**Resources:** Qiu-Yan Hu.

**Software:** Qiu-Yan Hu, Cheng-Zhi Xu, Jun Zhou.

**Supervision:** Cheng-Zhi Xu, Jun Zhou.

**Validation:** Cheng-Zhi Xu.

**Visualization:** Fei Yin.

**Writing – original draft:** Qiang Shi, Cheng-Zhi Xu, Fei Yin.

**Writing – review & editing:** Qiang Shi, Fei Yin.

## Supplementary Material


