# Supplementary material for: Association between ketogenic diet and cognitive function in older adults: The mediating role of neutrophil to high-density lipoprotein cholesterol ratio
Source: Medicine (Baltimore). 2026 Jan 30;105(5):e47441. doi: 10.1097/MD.0000000000047441 (PMC12863830; doi:10.1097/MD.0000000000047441)
Supplement: Supplementary file 1 [file medi-105-e47441-s001.pdf]

| Covariate Category | Covariate Name | Categories or source |
|---|---|---|
| Demographic Variables | Gender | Male, Female |
| | Age | From survey questionnaire inquiries |
| | Race | Mexican American, Other Hispanic, Non-Hispanic White, Non-Hispanic Black, Other Race (including multi-racial) |
| | Educational Level | Less than high school, High school graduate/GED or equivalent, Higher than high school |
| | Household Poverty-to-Income Ratio (PIR) | ≤1.3, 1.3–3.5, >3.5 |
| | Marital Status | Married/Living with a partner, Widowed/Divorced/Separated, Never married |
| Life behavior and disease Variables | Body Mass Index (BMI) | <25 kg/m², 25-30 kg/m², ≥30 kg/m² |
| | Smoking Status | Nonsmokers (<100 cigarette in lifetime), Smokers (>100 cigarette in lifetime) |
| | Drinking Status | Nondrinkers (<12 drinks in a year), Drinkers (achieved 12 drinks in a year) |
| | Diabetes and hypertension | From the medical history obtained in survey questionnaire inquiries |
| | Physical activity | Yes [achieved 600 metabolic equivalent (MET) minutes per week], No [<600 metabolic equivalent (MET) minutes per week] |
| | Energy intake | The average of the intake from two dietary surveys |

**Supplement Table 1**. Classification and definition of covariates.

| | CREAD scores | | AFT scores | | DSST scores | | Summary Z scores | |
|---|---|---|---|---|---|---|---|---|
| | β (95% CI) | *P* | β (95% CI) | *P* | β (95% CI) | *P* | β (95% CI) | *P* |
| Continuous | 2.75 (0.47, 5.04) | 0.018 | 0.96 (-0.96, 2.88) | 0.328 | 6.63 (1.72, 11.53) | 0.008 | 0.42 (0.14, 0.70) | 0.004 |
| Q1 | | | | | | | | |
| Q2 | -0.15 (-0.84, 0.54) | 0.670 | -0.08 (-0.67, 0.50) | 0.775 | 0.89 (-0.60, 2.37) | 0.241 | 0.03 (0.06, 0.11) | 0.543 |
| Q3 | 0.51 (-0.19, 1.21) | 0.156 | -0.19 (-0.78, 0.40) | 0.53 | 0.58 (-0.93, 2.08) | 0.455 | 0.04 (-0.05, 0.12) | 0.412 |
| Q4 | 0.86 (0.15, 1.56) | 0.017 | 0.37 (-0.22, 0.97) | 0.216 | 2.40 (0.88, 3.91) | 0.002 | 0.15 (0.06, 0.23) | 0.001 |
| *P* for trend | 0.004 | | 0.210 | | 0.003 | | < 0.001 | |

**Supplement Table 2**. The associations of DKR and cognitive function in sensitivity analysis.

Models were adjusted for age, gender, race, education, PIR, marital status, BMI, smoking status, drinking status, diabetes, hypertension, activity, energy intake and depression.
